# OA14.01. Relaxation-induced anxiety: predictors and subjective explanations among young adults

**DOI:** 10.1186/1472-6882-12-S1-O53

**Published:** 2012-06-12

**Authors:** C Luberto, S Cotton, A McLeish

**Affiliations:** 1 University of Cincinnati, Cincinnati, USA

## Purpose

Relaxation is used to treat multiple physical and mental health conditions. However, with 17% to 53% of adults reporting relaxation-induced anxiety (RIA), relaxation treatments may not be suitable for all individuals. Indeed, RIA is associated with poor treatment outcomes (e.g., increased panic attack frequency). Therefore, understanding the characteristics of and reasons for the fear of relaxation will help determine how to administer relaxation treatments safely and appropriately. This study used a mixed methods approach to examine predictors and explanations for RIA.

## Methods

Participants were 300 undergraduate students (mean age=21.25; 73% female; 83% white) who completed self-report measures assessing their health and emotions. RIA was measured using a single dichotomous item, “Do you ever feel anxious when doing relaxing activities like yoga, meditation, or getting a massage?” (Y/N). Those who reported ‘yes’ were asked, “What about these activities makes you anxious?” (open-ended). Chi-square and t-tests were used to compare demographic variables and self-reported history of medical and psychiatric problems between those who did and did not endorse RIA. Multivariable binary logistic regression was used to model the effects of significant (p<.05) clinical and demographic variables. Qualitative responses were examined for explanatory themes.

## Results

Approximately 15% (n=46) of individuals reported RIA. RIA was associated with a self-reported history of asthma, insomnia, depressive symptoms, social anxiety, and generalized anxiety (χ2(1)=5.17 to 20.71, p<.05 to p<.001), but not with demographic variables. In multivariable analyses, asthma (OR=2.38, p<.05) and generalized anxiety (OR=4.09, p<.05) remained significant predictors. Reported themes regarding reasons for RIA included restlessness, boredom, embarrassment, unwanted cognitive activity, and worry regarding the ability to relax (e.g., “I should feel calm but I don’t”). Findings suggest that a self-reported history of asthma and anxiety, particularly generalized anxiety disorder, are associated with adverse reactions to relaxation. Worries regarding the relaxation procedure itself should be addressed prior to administering treatment.

## Conclusion

A self-reported history of asthma and anxiety, particularly generalized anxiety disorder, are associated with adverse reactions to relaxation. Worries regarding the relaxation procedure itself should be addressed prior to administering treatment.

